# Synthesis and optimization of chitosan nanoparticles: Potential applications in nanomedicine and biomedical engineering

**Published:** 2014

**Authors:** Arezou Ghadi, Soleiman Mahjoub, Fatemeh Tabandeh, Farid Talebnia

**Affiliations:** 1 Faculty of Chemical Engineering, Noshirvani University of Technology, Babol, Iran.; 2 Fatemeh Zahra Infertility and Reproductive Health Research Center, Babol University of Medical Sciences, Babol, Iran.; 3 Department of Biochemistry & Biophysics, Faculty of Medicine, Babol University of Medical Sciences, Babol, Iran.; 4 National Institute of Genetic Engineering and Biotechnology (NIGEB), Tehran, Iran.

**Keywords:** Nanomedicine, Nanoparticle, Chitosan, Biomedical engineering.

## Abstract

***Background: ***Chitosan nanoparticles have become of great interest for nanomedicine, biomedical engineering and development of new therapeutic drug release systems with improved bioavailability, increased specificity and sensitivity, and reduced pharmacological toxicity. The aim of the present study was to synthesis and optimize of the chitosan nanoparticles for industrial and biomedical applications.

***Methods:*** Fe_3_O_4_ was synthesized and optimized as magnetic core nanoparticles and then chitosan covered this magnetic core. The size and morphology of the nano-magnetic chitosan was analyzed by scanning electron microscope (SEM). Topography and size distribution of the nanoparticles were shown with two-dimensional and three-dimensional images of atomic force microscopy (AFM). The nanoparticles were analyzed using transmission electron microscopy (TEM).

***Results:*** The chitosan nanoparticles prepared in the experiment exhibited white powder shape. The SEM micrographs of the nano-magnetic chitosan showed that they were approximately uniform spheres. The unmodified chitosan nanoparticles composed of clusters of nanoparticles with sizes ranging from 10 nm to 80 nm. AFM provides a three-dimensional surface profile. The TEM image showed physical aggregation of the chitosan nanoparticles.

***Conclusion: ***The results show that a novel chitosan nanoparticle was successfully synthesized and characterized. It seems that this nanoparticle like the other chitosan nano particles has potential applications for nanomedicine, biomedical engineering, industrial and pharmaceutical fields.

In recent years, the identification of the unique properties of nanoparticles has allowed their application in many fields such as biomedicine, drug and gene delivery, tumor detection, tumor vaccines, tissue engineering, MRI contrast enhancement, sensor development, fluorescent biological labels, detection of pathogens, detection of protein, separation and purification of cells and biological molecules, probing of DNA structure, environmental remediation and water purification (1-7). They are used in the diagnosis and therapeutics due to their unique properties of having small size, large surface area to volume ratio, stability over high temperatures, translocation into the cells and high reactivity to the living cells. They are available in different sizes and shapes due to their ability to react and agglomerate with other nanoparticles in their surroundings.

They also exhibit exceptional optical properties making them capable of producing quantum effects suitable for imaging applications (1-3). Most commonly studied metallic nanoparticles include gold, silver, aluminum, zinc, iron and titanium oxide nanoparticles (8). However, due to their “nano” size, their entry is easily facilitated into different cells posing one of the greatest problems in using these nanoparticles for targeted delivery to specific tissues. To resolve this problem, researchers have been conjugating these nanoparticles with various biomolecules and ligands to develop strategies for targeted delivery. The large surface-area-to-volume ratio of a nanoparticle allows it to serve as an efficient carrier of biomolecules. This feature has resulted in the development of many biomolecule-nanoparticle (bio-NP) hybrids for biomedical applications in the diagnosis and localized treatment of diseases. The benefits of magnetic nano-carriers are resistant against chemical and microbial environment, high mechanical resistance and good thermal stability (8-10).

Chitosan [(1, 4)-2-amino-2-deoxy-D-glucan] is a linear polyaminosaccharide obtained by N-deacetylation of chitin, the second most abundant natural bio-polymer, after cellulose. It can be easily processed in diverse forms, such as films, threads, tablets, membranes and microparticles/ nanoparticles, allowing the design of a variety of medical and pharmacological devices adaptable to end purposes. In particular, in medicine, chitosan may be useful in bandages to reduce bleeding and as an antibacterial agent can also be used to help deliver drugs through the skin. It is also used in the development of chitosan drug control releasing systems including chitosan sponges, chitosan film, chitosan beads, chitosan microbeads (microspheres) and chitosan nanoparticles. In other words, nano-magnetic is the core and chitosan polymer covering the magnetic cores. The process is called coating. These magnetic nanoparticles can be used as powerful carriers for enzyme immobilization (11, 12).

Chitosan nanoparticles have an antitumor role through improving the body’s immune function (13-18). Despite the well known advantages of exploiting chitosan in these fields, an additional work needs to be done to optimize chitosan’s formulations and enhance its physicochemical properties for different uses. The aim of the present study was to synthesis and optimize the formulation parameters of chitosan magnetic nanoparticles for biomedical engineering and pharmaceutical applications and present a method for synthesizing of novel chitosan magnetic nanoparticles. 

## Methods

This experimental study was done in the Biochemistry Department of Babol University of Medical Sciences, Babol and National Institute of Genetic Engineering and Biotechnology, Tehran, Iran. 

**Synthesis and optimization of magnetic core nanoparticles: **In this study, Fe_3_O_4_ was synthesized and optimized as magnetic core nanoparticles. For the preparation of magnetic core nanoparticles, aqueous solutions of Fe (II) /Fe (III) chloride (molar ratio 1:2) was prepared and added to an oxygen free container while N_2_ was flown in it. The solution was stirred for 30 sec and then, 20 mL NH_3_ was quickly added to the solution and pH was adjusted at 9. After 45 min, the prepared nanoparticles were stuck to the magnet and separated easily. The synthesized magnet nanoparticles were washed with deionized water at pH7, and dried at 37^°^C. The amount of 0.02 g magnet nanoparticles were dissolved in 50 mL deionized water and combined with 50 mL tri-sodium citrate solution. The result solution was sonicated for 30 min. This procedure was confirmed after optimization of the method.

**Synthesis** **and optimization of chitosan nanoparticles: **The amount of 500 mg of chitosan (medium molecular weight and 85% deacetylated, Sigma Chemical, St. Louis, USA) was dissolved in 50 ml of 1% acetic acid solution and stirred at 1000 rpm for 25 min at room temperature until the solution became clear. The resulting solution was sonicated and then titrated by addition of NaOH or HCL solution adjusted to pH5 and filtered using 0.2µ mesh. For coating process, 5 ml nano-magnetic solution was added to 75 mL deionized water and sonicated for 10 min. Then, chitosan solution was added and sonicated for 5 min. The resulting solution was clear. 

**Electron microscopy analysis: **After the preparation of the synthesized chitosan nanoparticles, the characterization of the nanoparticle was examined by scanning electron microscope (SEM) using Stereoscan (model S360 brand SEM – Leica Cambridge, Cambridge, UK). Also, the size and morphology of the nano-magnetic chitosan was observed by transmission electron microscopy (TEM) (Philips CM200 EFG, FEI Company, Eindhoven, Netherlands). In addition, the pictures of atomic force microscopy (AFM) were taken using an Autoprobe CP Research AFM system (model AP- 2001, Thermomicroscopes, USA). 

## Results

**Figure. 1:**
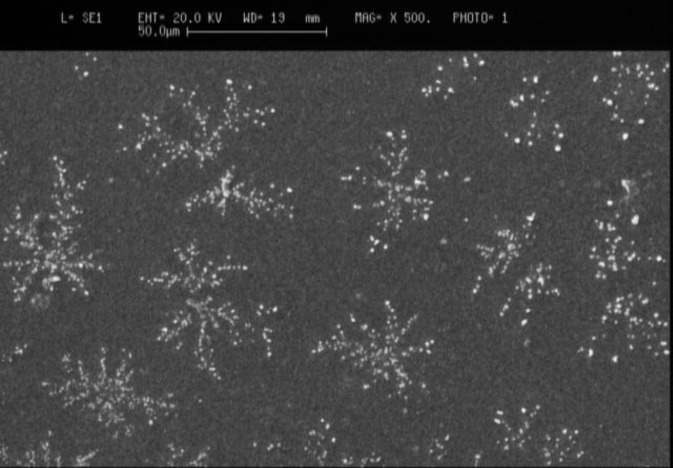
Scanning electron microscopy (SEM) micrograph of the chitosan nanoparticles

Chitosan nanoparticles prepared in the experiment exhibited white powder shape. The morphology of chitosan nanoparticles was analyzed by scanning electron microscope (SEM). The unmodified chitosan nanoparticles composed of clusters of nanoparticles (figure 1).

**Fig. 2:**
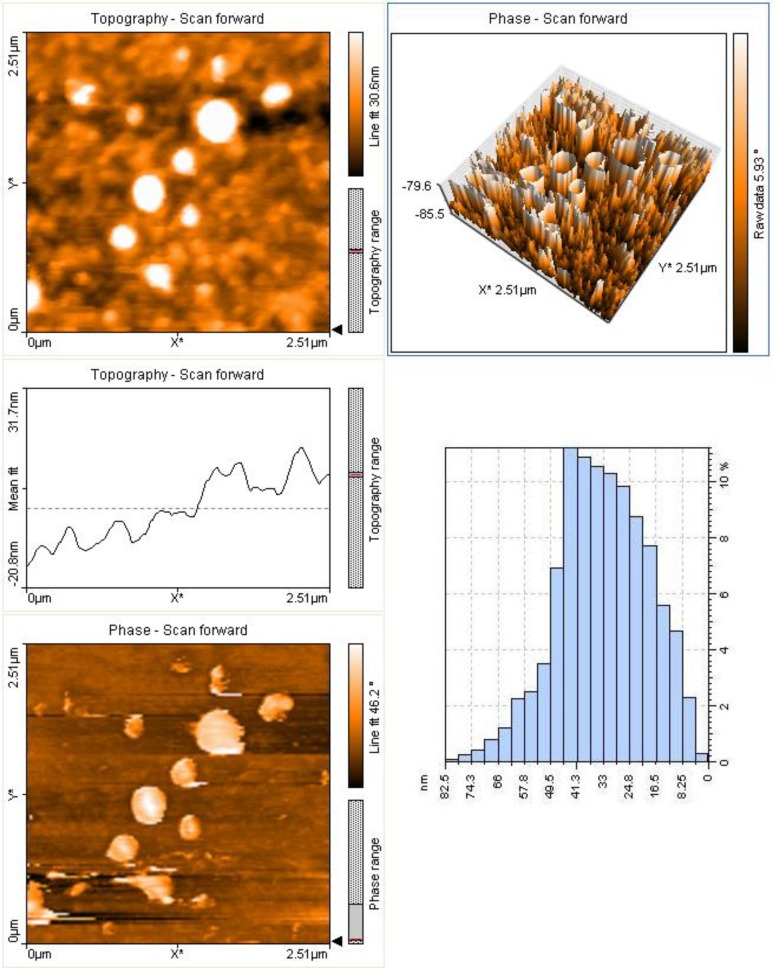
Topography and size distribution of chitosan nanoparticles using Atomic Force Microscopy a) two-dimensional, b) three-dimensional c) size distribution

The scanning electron micrographs of the chitosan magnetic nanoparticles showed that they were approximately uniform spheres**. **Unlike electron microscopes, which provide a two-dimensional projection or a two-dimensional image of a sample, AFM provides a three-dimensional surface profile. Although the lateral dimensions are influenced by the shape of the probe, the height measurements can provide the height of nanoparticles with a high degree of accuracy and precision. Figure 2 showed that the analysis of size distribution of the chitosan nanoparticles by atomic force microscopy. As shown in the histogram of AFM (Fig 2-C), the size distribution of the nano-magnetic chitosan particles is between 10-80 nm.

**Figure. 3:**
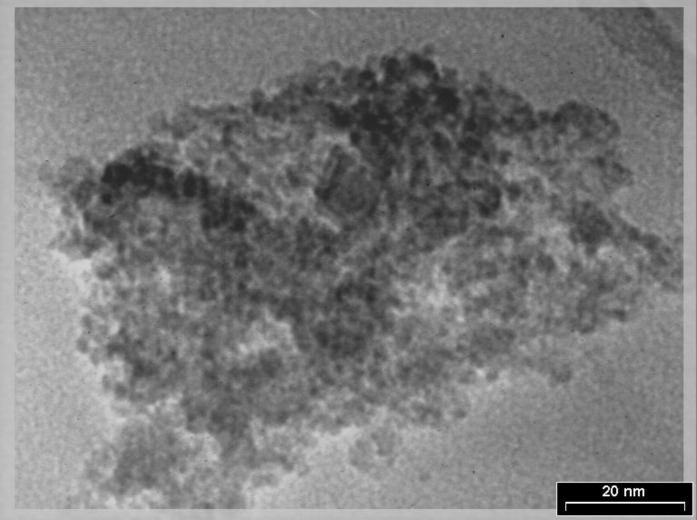
Transmission electron microscopy (TEM) of the chitosan nanoparticles

The observation from transmission electron microscope (TEM) gave us information on the particle shape and the determination of particle size. Typical TEM micrograph of the chitosan nanoparticles was shown in figure 3. 

In the present study, TEM images showed physical aggregation of the chitosan nanoparticles. According to electron microscope, the analysis of the chitosan magnetic nanoparticles diameter of the essentially monodispersed Fe3O4 particles ranged between 5 am to 8 nm, and the diameter of the nano-magnetic chitosan ranged between 10 nm to 80 nm. The Fe3O4 particles were well-coated by chitosan.

## Discussion

In the present study, we successfully synthesized and optimized a novel magnetic chitosan nanoparticle as potential applications for medical, pharmaceutical and industrial fields. The applications of magnetic nanoparticles in medical and pharmaceutical fields can be rationalized as follows: first, the magnetic chitosan nanoparticles are a class of intrinsically ordered magnetic materials. As their magnetic signal is generated by the application of external strong magnetic field, it far exceeds that signal from any of the known molecules. This makes them readily identified by a magnetic sensor or magnet from other molecules. Second, their capability of being manipulated under an external magnetic field provides controllable means of magnetically tagging biomolecules, leading to potentially highly sensitive biosensing highly efficient bioseparation, and magnetic resonance imaging (MRI) contrast enhancement, as well as site-specific drug delivery (5, 19, 20). 

Chitosan nanoparticles have become of great interest as polymeric platforms for the development of new pharmacological and therapeutic drug release systems with improved biodistribution and increased specificity and sensitivity, and reduced pharmacological toxicity. Chitosan nanoparticles have been found appropriate for non-invasive routes of drug administration: nasal, oral, ocular and pulmonary routes. These applications are facilitated by the absorption-enhancing effect of chitosan. Additionally, chitosan nanoparticles have been proposed as non-viral vectors in gene therapy and have shown adjuvant effect in vaccines. Absorption and bioavailability of drug encapsulated into chitosan nanoparticles can be improved, so they can be used to deliver gene drugs, protein drugs and other compounds and can protect them effectively from enzyme degradation in vivo (21, 22). 

As a carrier, nanochitosan can improve DNA and drug bioavailability, resistance in vivo to enzyme drop solutions, enhance controlled sustained release of biomaterials, reduce toxicity, and be prepared under mild conditions without the use of an organic solvent, thereby avoiding DNA and drug destruction, as well as preventing residual solvent remaining after the preparation process (23). 

Chitosan nanoparticles can be used to alter protein loading and adjust the value of each parameter during preparation. They also have high stability, high protein packing efficiency, can be prepared as a lyophilized powder, and are easy to store and transport (24). In our study, we prepared the magnetic chitosan nanoparticles as white powder and confirmed characteristics of the nano product using scanning electron microscope, transmission electron microscopy, and atomic force microscopy. The nanoparticles were stored as lyophilized powder for various applications. Most biological macromolecules, including proteins, cannot enter cells because of the selective permeability of the cell membrane, which hampered application of tumor vaccines (25). Conventional colloidal carriers are rapidly removed from the bloodstream by the reticuloendothelial system (RES), which is a part of the mononuclear system (MPS) after intravenous administration. Nanoparticulate systems have been used to improve the blood circulation time and tumor targeting efficacy, because the tumor vascular permeability allows the penetration of particles up to 400 nm in diameter. According to our study, the unmodified chitosan nanoparticles composed of clusters of nanoparticles with sizes ranging from 10 nm to 80 nm. 

The antitumor efficacy of chitosan nanoparticles administered by intravenous injection is probably attributed to their little particle size. Particle size was proven as an important feature related to obtaining optimal in vitro efficacy of chitosan nanoparticles. Particle size also had a crucial impact on the in vivo fate of a drug delivery system. Decreasing particle size could increase the surface-to volume ratio and specific surface area, which could increase the dissolution and thus increase the bioavailability of poorly water soluble molecules. The smaller size particles seem to have efficient interfacial interaction with the cell membrane compared to larger size particles due to the endocytosis of small size particles. Small size particles could improve the efficacy of the particle-based oral drug delivery systems. Also, the use of particle size reduction to increase the oral bioavailability of drugs has been obtained. Nanoparticles can prolong the blood half-life of drugs and increase the efficacy by intravenous injection (26, 27). 

The major disadvantage of most drugs for tumor chemotherapy is their relative nonspecificity. The drugs are administered intravenously for general system distribution, resulting in deleterious side effects as they attack normal, healthy cells in addition to the target tumor cells. Preferably, the drugs should be localized to the tumor site. Chitosan nanoparticles are now being modified for sustained/ controlled release and targeting. The attachment of drugs to magnetic particles can be used to reduce drug dose and potential side effects on healthy tissue and the costs associated with drug treatment. The size, charge and surface chemistry of the magnetic particles are particularly important in affecting both blood circulation time as well as the bioavailability of the particles within the body (4, 13, 18, 19). In the absence of any surface coating, nano-magnetic particles have hydrophobic surfaces with a large surface area to volume ratio. Due to hydrophobic interactions between the particles, they tend to agglomerate forming large clusters. In the present study, the TEM image showed the physical aggregation of the nanomagnetic chitosan. There are two possible reasons for this behavior. First, as the Fe3O4 nanoparticles were present in the pores of the chitosan nanoparticles, this may be cross-link between chitosan chains which caused the aggregation of magnetic nanoparticles. Second, the diameter of the particles was too small, and the surface energy was very high, which may cause the physical aggregation. Although some observed aggregation could be caused by drying during TEM sample preparation.

In our study, Fe_3_O_4_ was synthesized and optimized as magnetic core nanoparticles and then chitosan covered this magnetic core as a shell. We showed the high magnetic characteristics of the synthesized nano chitosan. Magnetic nanoparticles respond resonantly to an alternating magnetic field, allowing the transfer of magnetic energy to the particles as a form of heat. This has been proposed to be one of the key approaches to successful cancer therapy in the future (28). Also, nanomaterials combined with tumor antigens allow tumor vaccines to have stronger biocompatibility, permeability, and targeting properties (1). In the future, nanotechnology makes more progress, and more new nanomaterials continue to appear, they will be used increasingly in the fields of tumor vaccines, gene and drug carriers. Finally as a conclusion, we successfully synthesized and characterized a novel chitosan nanoparticle using the optimization of condition. It is believed that the synthesized novel chitosan nanoparticles can be considered for different biomedical applications.

## Notes

**Funding: **This study was funded by a grant from Noshirvani University of Technology, Babol and National Institute of Genetic Engineering and Biotechnology (NIGEB), Tehran Iran.
